# Acute Myeloid Sarcoma: An Unusual Cause of Diarrhea

**DOI:** 10.7759/cureus.10748

**Published:** 2020-10-01

**Authors:** Danish Safi, Luna Acharya, Maimoona Khan, Oana Paun, Carlos Vigil

**Affiliations:** 1 Hematology and Medical Oncology, West Virginia University, Morgantown, USA; 2 Internal Medicine, University of Iowa Hospitals and Clinics, Iowa City, USA; 3 Internal Medicine, Shifa International Hospital Islamabad, Islamabad, PAK; 4 Internal Medicine/Hematology-Oncology, University of Iowa, Iowa City, USA; 5 Internal Medicine/Hematology-Oncology, University of Iowa Hospitals and Clinics, Iowa City, USA

**Keywords:** diarrhea, acute myeloid sarcoma

## Abstract

A 62-year-old man with a past medical history of hypothyroidism was admitted for diarrhea and abdominal pain for three weeks. Initial workup for diarrhea was negative. His condition deteriorated after hospitalization. He underwent sigmoidoscopy which showed rectosigmoid mucosal ulceration. Pathology showed leukemic cells infiltration of the mucosa. The patient underwent bone marrow biopsy which confirmed the diagnosis of acute myeloid leukemia (AML). He received induction chemotherapy and his symptoms improved.

## Introduction

Diarrhea can be an unusual presentation of acute myeloid sarcoma. Acute myeloid sarcoma mostly presents with signs and symptoms of gastrointestinal obstruction but in our case, it presented with diarrhea which is a very rare presentation and has been presented very rarely in the past. Extensive workup helped in early diagnosis and treatment, reducing morbidity and mortality.

## Case presentation

A 62-year-old male, with a 30-year smoking history and a past medical history of hypothyroidism, presented with 10-12 episodes per day of watery, nonbloody diarrhea for three weeks, along with subjective fevers. The patient reported that he had an upper respiratory tract infection three weeks prior, for which he received five days of azithromycin and prednisone. The patient denied abdominal pain, nausea, vomiting, melena, and weight loss. On examination, he was afebrile and hemodynamically stable. On abdominal examination, there was no abdominal tenderness and no hepatosplenomegaly.

**Figure 1:**
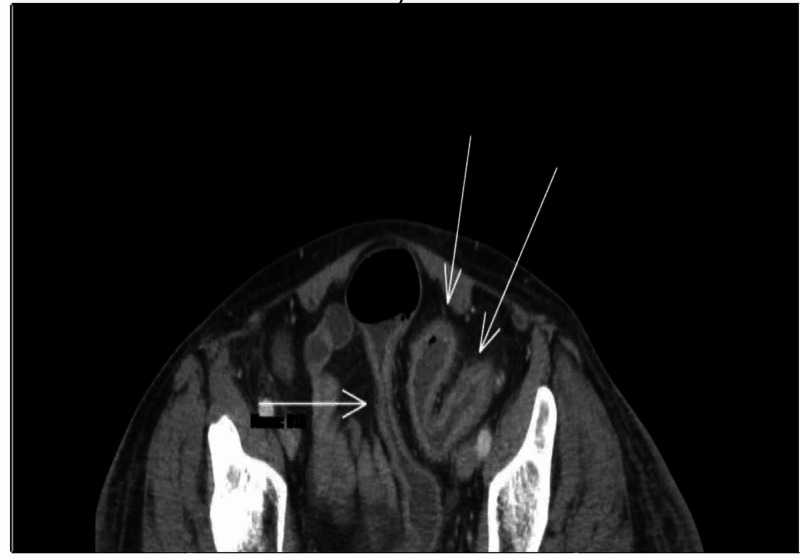
CT scan of abdomen and pelvis showing colitis extending from the hepatic flexure to the rectum. Arrows show mild wall thickening of the sigmoid colon with mucosal hyper-enhancement, mild surrounding fat stranding, and mesenteric hyperemia.

**Figure 2:**
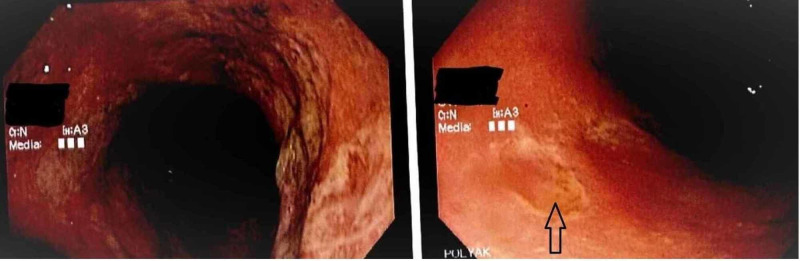
Flexible sigmoidoscopy showed multiple punched-out ulcerations throughout the rectosigmoid colon with white fibrous centers. Sigmoidoscopy showed several punched-out ulcer.

**Figure 3:**
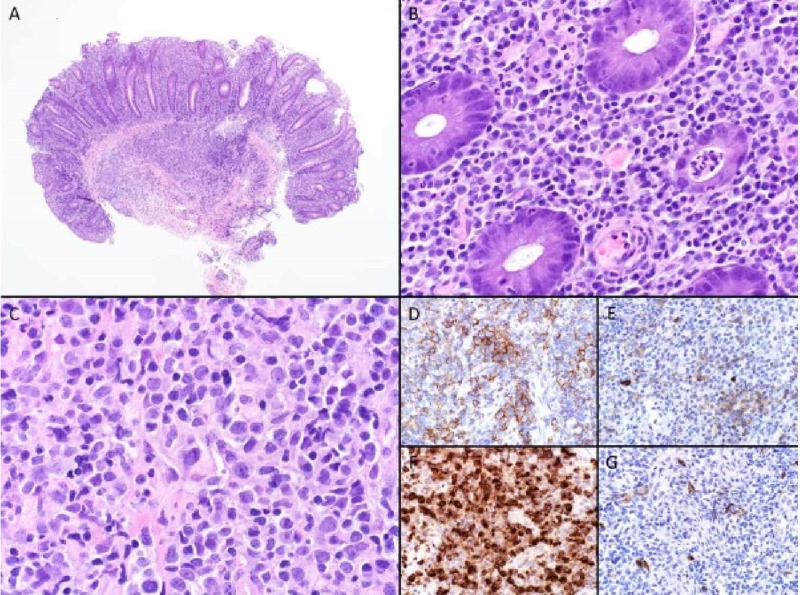
IHC showed CD20 and CD3 positive, atypical infiltrate expressing CD43 (diffuse) with subpopulations expressing kit and CD68 and no significant expression of CD34. Colonic involvement by AML (granulocytic sarcoma): A. Low power photomicrograph highlights inflammatory infiltrate centered on the lamina propria and extending into submucosa (H&E 40x). B. High power photomicrograph highlights lamina propria infiltrate composed mainly of plasma cells and cryptitis and a crypt abscess (i.e., chronic active colitis pattern of injury) (H&E 400x). C. In several areas, predominantly submucosal, there is an infiltrate of large cells with blastic chromatin, folded nuclear contours, and fairly abundant, delicate, pale eosinophilic cytoplasm (H&E 400x). These cells expressed D. CD43 (multifocal), E. KIT (focal), F. lysozyme (diffuse), and not G. CD34, supporting the diagnosis of colonic involvement by AML (immunoperoxidase, each 400x). IHC, immunohistochemistry; AML, acute myeloid leukemia

Laboratory studies were significant for leukocytosis of 48.4 k/mcL with left shift, but no bandemia. His comprehensive metabolic panel was normal. The stool osmolol gap was <50 mOsm/kg, supporting secretory diarrhea. Infectious workup was negative for Clostridium difficile, ova/parasite, cytomegalovirus (CMV), herpes simplex virus (HSV), Adenovirus, Spirochete, cryptococcal, microsporidia, and human immunodeficiency virus (HIV). A CT scan of the abdomen and pelvis showed colitis extending from the hepatic flexure to the rectum (Figure 1). The patient was started on loperamide but continued to have diarrhea. Flexible sigmoidoscopy showed multiple punched-out ulcerations throughout the rectosigmoid colon with white fibrous centers (Figure 2). Biopsy showed colonic involvement by granulocytic sarcoma (acute myeloid leukemia, AML) with severe active colitis with ulceration and small adherent pseudomembrane. Histology report showed a heterogeneous population of inflammatory cells within the lamina propria, including scattered mast cells (CD117+), histiocytes (Muramidase+), and lymphoid cells (PU-1+). Immunohistochemistry (IHC) showed CD20 and CD3 positive, atypical infiltrate expressing CD43 (diffuse) with subpopulations expressing kit and CD68, and no significant expression of CD34 (Figure 3). Bone marrow aspirate and biopsy showed hypercellular marrow (80%) with 55% blasts, chromosomes [46, XY]. Fluorescence in situ hybridization (FISH) (the chromosome abnormalities characteristic for AML, such as trisomy 8 and deletions of chromosome 5q, 7q, 11q, as well as chromosome rearrangement involving RUNX1T1/RUNX1, PML/RARA, CBFB, and MLL regions were not present), molecular genetics [FLT3 both alleles (ITD and TKD) negative and NPM1 positive mutation] supported the diagnosis of AML.

Diarrhea can be due to many reasons. Our main differential was infection and inflammation, which were ruled out with thorough investigations. The patient was treated with induction chemotherapy with 7+3 [cytarabine (100 mg/m2)/daunorubicin (60 mg/m2)]. A repeat bone marrow biopsy showed a hypocellular marrow (<5%) with 1% blasts. Also repeat colonic biopsy showed no evidence of ulcers and granulocytic sarcoma. The patient’s diarrhea did improve with induction chemotherapy. The patient is alive and still living a healthy life with current outpatient follow-up.

## Discussion

Acute myeloid leukemia is characterized by the accumulation of ≥20% myeloid premature blast cells in the bone marrow and they are most often found in the peripheral blood [1]. AML patients most commonly present with abnormal hematologic laboratory results, including leukocytosis, anemia, and thrombocytopenia. The diagnostic workup of AML involves the integration of history, clinical presentation, and laboratory results with studies performed by a pathologist [2]. Less than 1% of patients with AML will present with prominent extramedullary disease i.e., myeloid sarcoma or granulocytic sarcoma or chloroma [3]. Myeloid sarcoma can rarely involve the gastrointestinal tract and can present with various symptoms like bleeding, perforation, abdominal pain, obstruction, intussusception, infarction of the liver, bile duct obstruction, pancreatitis, appendicitis, and portal hypertension [4]. CT imaging can show various findings like single or multiple mass obstructing the lumen of the gut, or exophytic lesions involving the peritoneum [5]. Myeloid sarcoma has shown to involve various parts of the gastrointestinal tract like the oral cavity [6], esophagus, stomach, jejunum [7], gallbladder, bile ducts, pancreas [8], colon [9], liver [10], appendix [11], and anal region [12]. Our patient presented with watery diarrhea and it is uncommon for myeloid sarcoma to present with common gastrointestinal symptoms [12]. He was diagnosed with leukocytosis during an infectious workup. Along with a workup for cancer, we decided to do a biopsy of the colon to exclude inflammatory causes of diarrhea i.e., inflammatory bowel disease (IBD). Myeloid sarcoma poses a significant diagnostic challenge, and biopsy and staining with immunohistochemical markers play a vital role in diagnosis. The immunophenotype is characteristic based on whether myeloid sarcoma is granulocytic (MPO+, lysozymes+, CD34+/−), monoblastic (MPO−, CD68+, lysozyme−, CD34+), myelomonoblastic (MPO+/−, CD68+, lysozyme+/−, CD34+/−), megakaryoblastic (Factor VIII+, CD31+), or erythroblastic variant (glycoprotein c+) [10]. It is difficult to distinguish myeloid sarcoma from large cell non-Hodgkin’s lymphoma [12-13]. Our patient had CD34 negative myeloid sarcoma, which is a rare entity [14]. Current treatment for myeloid sarcoma is induction chemotherapy with or without radiotherapy. Our patient received induction chemotherapy, and a repeat bone marrow biopsy and colonoscopy revealed no evidence of AML. His diarrhea was completely resolved with induction chemotherapy.

## Conclusions

It is unusual to find a diagnosis of myeloid sarcoma in patients who present with chronic diarrhea. Our case illustrates the need for detailed evaluation for chronic diarrhea in patients with concern for underlying hematologic malignancy.
